# Comparative Analysis of Majority Language Influence on North Sámi Prosody Using WaveNet-Based modeling

**DOI:** 10.1177/0023830920983591

**Published:** 2020-12-29

**Authors:** Katri Hiovain, Antti Suni, Sofoklis Kakouros, Juraj Šimko

**Affiliations:** University of Helsinki, Finland

**Keywords:** Dialectal variation, embeddings, North Sámi, prosodic typology, WaveNet

## Abstract

The Finnmark North Sámi is a variety of North Sámi language, an indigenous, endangered minority language spoken in the northernmost parts of Norway and Finland. The speakers of this language are bilingual, and regularly speak the majority language (Finnish or Norwegian) as well as their own North Sámi variety. In this paper we investigate possible influences of these majority languages on prosodic characteristics of Finnmark North Sámi, and associate them with prosodic patterns prevalent in the majority languages. We present a novel methodology that: (a) automatically finds the portions of speech (words) where the prosodic differences based on majority languages are most robustly manifested; and (b) analyzes the nature of these differences in terms of intonational patterns. For the first step, we trained convolutional WaveNet speech synthesis models on North Sámi speech material, modified to contain purely prosodic information, and used conditioning embeddings to find words with the greatest differences between the varieties. The subsequent exploratory analysis suggests that the differences in intonational patterns between the two Finnmark North Sámi varieties are not manifested uniformly across word types (based on part-of-speech category). Instead, we argue that the differences reflect phrase-level prosodic characteristics of the majority languages.

## 1 Introduction

**Figure 1.**
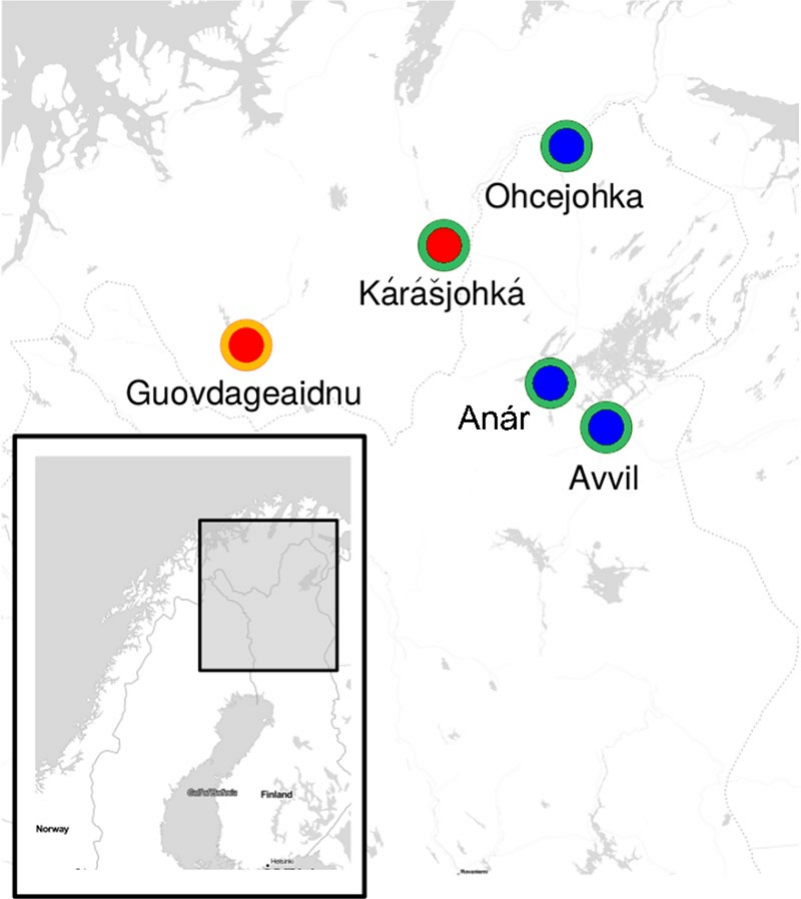
The villages in the Finnmark North Sámi traditional speaking area. The orange circle marks the western dialect, green circle the eastern dialect, red circle shows the villages in Norway and blue circles the villages in Finland. Map compiled in RStudio (Allaire, 2012).See the online article for the color version of this figure. See the online article for the color version of this figure.

North Sámi is one of the nine currently spoken Sámi languages, forming the Sámi branch in the Uralic language family. It belongs to the Western group of the Sámi languages that are spoken in the northern parts of the Scandinavian peninsula (Aikio et al., 2015), as depicted in Figure 1. For comprehensive presentations of the Sámi languages and their structures, see Korhonen (1981), Kulonen et al. (2005), and Sammallahti (1998). Although North Sámi has the highest number of speakers of the Sámi languages (approximately 20,000–30,000; Kulonen et al., 2005) and is an official language in six northernmost counties in Norway and legally recognized in Sweden and Finland (Seurujärvi-Kari, 2012), it still remains a lesser-documented language and its constantly evolving spoken form would need more recent and more thorough documentation.

The North Sámi language is divided into three main dialect groups: Torne; Finnmark; and Sea. This paper focuses on the Finnmark dialect group spoken in the area spanning the northernmost parts of Norway and Finland. In parallel to the dialectal variability, the majority of state languages—Finnish and Norwegian—have a considerable influence on the spoken language of the areal varieties, presumably due to long and intensive language contact (Marjomaa, 2014). Many aspects of the language situation are reinforcing the majority language influence, for example the bilinguality of the speakers, the Sámi people moving away from their traditionally inhabited regions, and also the growing effect of digitalization (Aikio et al., 2015).

Stanford and Preston (2009) provide many examples of studies of phonetic and phonological variation of indigenous languages in connection with majority languages. Studies of Māori (Harlow et al., 2009) and Catalan (Montoya-Abat, 2009), for example, indicate that language contact is an inevitable aspect of research in indigenous and/or minority languages. Besides a variation on segmental level, studies have also documented prosodic features and their variation in minority languages such as Paraguayan Guaraní (Clopper & Tonhauser, 2013), Djambarrpuyŋu (Jepson, 2019) and Lakota (Mirzayan, 2010).

Recently, machine learning techniques have been applied to explore the North Sámi areal varieties, with particular attention paid to the influence of majority languages. Jokinen et al. (2016) used i-vector techniques to learn classification of varieties by the predominant majority language based on acoustic, segmental, and phonotactic features. Kakouros et al. (2020) investigated the influence of purely prosodic features: energy; fundamental frequency (F0); spectral tilt; duration; and their combinations. In Hiovain et al. (2018), a technique using wavelet decomposition of signals carrying purely prosodic information (F0 contour and energy envelope) showed a greater similarity of varieties spoken in the same country compared to the varieties exposed to the influence of another majority language.

By clustering the read speech North Sámi data from different areal varieties by majority language these studies have shown the presence of a measurable influence of the majority language on several phonetic aspects of the spoken language, including prosody. These machine-learning techniques, however, have not provided qualitative information regarding the manifestation of the differences underlying these influences. The primary aim of the present work is to expand the machine learning approach and identify at least some of the prosodic features that clearly reflect the majority language influence. The guiding hypothesis is that the local varieties of North Sámi to some extent incorporate some of the intonational characteristics of the respective majority languages, and that the perceived differences reflect the differences between Norwegian and Finnish in terms of prosody.

Figure 1 shows the places of origin of the participants whose recordings are analyzed in this study. The Guovdageaidnu and Kárášjohká varieties are spoken in Norway and have presumably been under the influence of Norwegian language, while Avvil, Anár, and Ohcejohka varieties are spoken in Finland and thus presumably exhibit Finnish influence in their prosodic characteristics.

## 2 Prosodic typology

Comparing prosodic characteristics of multiple languages is complicated because of the multidimensionality of the task. The comparisons need to span and combine suprasegmental (e.g., tonal and rhythmic properties) and the resulting intonation and stress patterns, as well as the presence and realization of word prosody (quantity contrast, pitch accent, etc.). Relatedly, development of a coherent prosodic typology is hampered by the lack of a language-independent prosodic transcription system (Hirst & Di Cristo, 1998; Jun, 2006).

Multiple existing studies have tried to rectify this shortcoming by adjusting the influential autosegmental–metrical (AM) modeling approach (Arvaniti, 2017; Ladd, 2008; Pierrehumbert, 1980), and its best known application, the tones and break indices (ToBI) annotation system (Silverman et al., 1992; Wightman et al., 1992).

Originally developed for annotating American English prosody for use in speech technology, the ToBI annotation system has been adapted for many languages besides English, including, for example, Japanese (Venditti, 2005) or West Greenlandic (Arnhold, 2007). Importantly in the context of the present work, there are AM frameworks established for Finnish (Suomi et al., 2008) and Norwegian (Heggtveit & Natvig, 2004) but not for North Sámi.

Besides documenting and analyzing individual languages, the AM framework and ToBI annotation tools have also been used to compare the intonational patterns of different languages, for example, English and German (Grabe, 1997), and dialects or areal varieties of one language, for example Portuguese (Vigário & Frota, 2003) and Irish (Dalton & Ní Chasaide, 2005, 2007a, 2007b). Phonological comparison of the intonation of languages or dialects requires not only strictly comparable speech material but also appropriate and adapted ToBI guidelines for the particular research question. Recently, there have been, however, contributions towards developing cross-linguistically transparent and consistent prosodic annotation systems, based on the AM and ToBI frameworks (see e.g., Frota, 2016; Hualde & Prieto Vives, 2016). These contributions have aimed to address the difficulties of comparing global patterns of intonation in different languages, suggesting that complementary levels of prosodic representations (phonetic and phonological) could be added to prosodic analysis. One of the advantages of including these annotation levels is that they could serve as a starting point to a phonological analysis of lesser-documented languages before establishing a phonological analysis of a language. These approaches, however, would require a great amount of systematic and transparent manual annotation done by experts, which might not be always possible to obtain.

Grabe (1997) comparatively compared intonational patterns of English and German using appropriately chosen comparable speech material, and an AM system specially developed for this research and language pair. This work identified the same underlying tonal structures in these two languages in identical contexts.

In a comparative study of intonational phonology of four Irish areal varieties, Dalton & Ní Chasaide (2007b) investigated the differences in peak timing and nuclear targets. In order to elicit various kinds of sentence intonations, the analyzed speech data contained various types of sentences, declaratives, wh-questions and yes/no-questions. The analysis was carried out by using an adaptation of ToBI annotation (IViE; Grabe, 2004), specially developed to account for variation of within English dialects. The results suggest a clear North–South divide in terms of the nuclear patterns of the dialects, where Northern varieties show a prominent low-rise pattern while in the Southern varieties, there is predominantly a falling nuclear contour. The authors have also compared their results with data from English varieties (Grabe & Post, 2002), bringing up a hypothesis that the rising nuclei in varieties of English are an influence from Irish.

As these examples suggest, these intonational phonological approaches require considerable knowledge about the intonational structure of the language, something that is not present for the application of the WaveNet model to North Sámi. An alternative approach, widely used in phonetic research is to first use a measure of some relevant prosodic characteristic (e.g., an extent of F0 movement and duration of syllables), and then analyze the measured variables using an appropriate statistical modeling (perhaps with language or a variety as one of the independent variables). Probably the best known illustrations of this approach are varied applications of durational measures and comparative investigation of rhythmic properties of various languages, for example, Dauer (1983), Gil (1986), Hyman (2006), and Jun (2006).

This approach to some extent bypasses the requirement to draw an explicit line between phonetics and phonology prior to the investigation, and is thus less theory dependent and more data driven. A reasonably clear hypothesis of the nature of differences between the investigated languages or dialects needs to be, however, formulated as this hypothesis influences an appropriate choice of phonetic measures. Also, the results can, of course, be strongly influenced by the type of speech data used in the study and by potential speakers’ idiosyncrasies (see e.g., Beňuš & Šimko, 2012), so selection and control of the analyzed material remains important.

The final approach involves the use of machine learning techniques that can learn complex statistical models of language prosody directly from speech material. Cummins et al. (1999), for example, trained recurrent long short-term memory networks on F0 and energy contours using a speech corpus containing multiple languages and used the performance of the recognition models on different languages as a measure of inter-language distance. More recently, a deep network speech synthesis approach—very similar to the present work—was used to find mutual relationships between tonal varieties of Swedish and evaluate the dialectal differences in terms of geographical distribution (Suni et al., 2019). Several studies concentrating on North Sámi, cited above (Hiovain et al., 2018; Jokinen et al., 2016; Kakouros et al., 2020) are also examples of this approach.

The machine learning techniques further reduce the requirement to select an appropriate measure and statistical analysis suitable for the given hypothesis about the nature of differences between the investigated languages of varieties. This way, these techniques facilitate a more exploratory kind of analysis of speech material, and might thus be well suited for lesser resourced or investigated languages. Importantly, the lack of specific phonological knowledge regarding North Sámi is precisely why the WaveNet approach is very useful in addressing our research questions.

The price one pays for this, probably inevitably, is the relative obscurity of the obtained results. While the machine learning techniques might yield a meaningful clustering of languages or language varieties into typologically interpretable groups, they do not necessarily reveal the phonetic or linguistic sources of such clustering. In the present work we thus extend the machine learning approach with a phonetic analysis aimed at addressing this shortcoming.

## 3 This study

As mentioned earlier, the aim of this paper is to address the challenging task of measuring and describing the majority language influence on North Sámi varieties. By necessity, the nature of this study is exploratory, and its main contribution is, in our opinion, methodological.

While we hypothesize that there are discernible “borrowings” from the majority languages in terms of prosodic patterns, we are, at the onset, agnostic as to their nature. Broadly speaking, the areal varieties under comparison are very similar and mutually intelligible, so the prosodic differences can be expected to be subtle and thus easily overlooked by the intonational phonology labeling schemes unless first identified by other methods.

It is important to note that, as is common for many minority, endangered languages, collecting speech material tailor-designed for a particular typological hypothesis is not practicable for North Sámi. Consequently, the analyzed speech material consists of read declarative sentences (Wikipedia texts) where major differences in post-lexical movements in intonation contours across utterances are not expected. The majority of the phonological ToBI representations in neutral intonational phrases would be expected to repeat a L+H* pattern for each content word with peaks gradually declining towards the end of the phrase (Valikangas, 2002). This, alongside the lack of a precise assumption regarding the nature of the majority language influence, and of the common ToBI annotating scheme for languages involved makes the AM approach unsuitable at this stage.

Instead, we present a novel methodology combining a machine learning WaveNet-based modeling with a traditional phonetic analysis. The aim of this exploratory method is to identify the potential effects of the majority language in our speech material. The machine learning component of the method is designed to quantify the differences between prosodic patterns of portions of the material (lexical items) as uttered by speakers from Finland on the one hand, and speakers from Norway on the other hand.

Briefly, a word embedding layer is trained as a part of a WaveNet synthesizer trained on a prosodic signal derived from the original recordings. The word embedding layer learns numerical vector representation of lexical items present in the material, separately for the renderings by the Finnish–Sámi bilinguals and by the Norwegian–Sámi bilinguals. The core methodological assumption is that the distance between two embedding vectors for a given word, one for each majority language, can be associated with the difference in prosodic patterns potentially attributable to the majority language influence.

In order to test this assumption, we calculated simple characteristics of F0 contours over the lexical items (standard deviation and range), averaged them over each majority language group, and compared the differences between these averages to the distance between the embedding vectors. Our *methodological hypothesis* (MH) thus states that the embedding distance measure correlates with the differences between appropriate phonetic intonational measures.

Word-level prosody is known to be influenced, among other factors, by the word’s part-of-speech (POS) type. The verbs, for example, are often assigned a weaker accent compared to, for example, nouns (Arnhold et al., 2010; Schmerling, 1976). We therefore test the MH for different POS categories in addition to the individual, most frequent, lexical items.

It is important to state here that our choice of word (lexical item) as a unit on which to investigate the majority language influence is determined by the scope over which a signal processing network of a meaningful size can learn statistical dependencies, that is, by the network’s receptive field (see subsection 5.2 on network architecture). We by no means exclude the possibility of a manifestation of this influence on other units of prosodic hierarchy, including larger portions such as intonational phrases, grammatical clauses or entire sentences. In this work we attempt to separate this potential source of variation from the word-level influence by training a phrase embedding in parallel to the word-level one, also to address different focus conditions in a sentence.

Given our methodological hypothesis, it is plausible to assume that the (word-related) influence of the majority language is best manifested on those lexical items with the greatest embedding distance. This assumption yields the *typological hypothesis* (TH) as follows: the greater the differences as captured by the embedding distance, the more characteristics of the majority language prosody are transferred to the respective realizations of the given lexical items. We will test this hypothesis by visualizing and qualitatively analyzing the intonational contours for the words with the greatest embedding distance.

In what follows we summarize the known characteristics of North Sámi prosody as well as the most relevant prosodic features of Norwegian and Finnish. Subsequently, we will describe in detail the material and the methodology used in this study.

## 4 Prosodic characteristics of North Sámi, Finnish, and Norwegian

Until recently, phonetic and phonological research on the North Sámi language has focused mostly on its complex morphophonological features (Baal et al., 2012; Kahn & Valijärvi, 2017; Sammallahti, 1998), and on the cross-linguistically rare three-way quantity contrast, which means that there are three phonologically contrastive lengths for segments: short; long; and overlong (Hiovain et al., 2020; Magga, 1984; Sammallahti, 1998). Prosodic features related to tonality (tone or intonation) or stress (e.g., word stress) have attracted much less attention by researchers; to our knowledge, there are no experimental studies on the topic. The earliest descriptions on North Sámi intonation have been presented by Jernsletten (1974, 1990); these also seem to be the most recent, pre-experimental descriptions on the intonation patterns of North Sámi.

Finnmark North Sámi is traditionally divided into two subdialects: Western; and Eastern. The traditional dialectal boundaries (as explained by e.g, Sammallahti, 1998) do not follow the state borders; Western and Eastern dialects are spoken in both Norway and Finland (see Figure 1). However, the Western dialect seems to be more common in Norway, and the Eastern one in Finland (like also in our data). These varieties of the Finnmark North Sámi are mostly mutually intelligible, but since the Finnmark area is very sparsely populated, there is considerable dialectal variation in phonology, morphology and lexicon (see e.g., Palismaa & Eira, 2001; Sammallahti, 1998).

The dialectal differences within Finnmark North Sámi have been up to date primarily studied in terms of Western/Eastern dialect distinction. Finnmark North Sámi dialects differ in both segmental (Sammallahti, 1998), and suprasegmental features related to quantity (Hiovain & Šimko, 2019; Hiovain et al., 2020) (see also a possible source of dialect-dependent source of interaction between quantity and tonality reported by Hiovain et al., 2020).

North Sámi and Finnish are both Uralic languages and thus share some structural and lexical features, while Norwegian belongs to a distinct, Indo-European family. No pair of these three languages is mutually intelligible.

In North Sámi and Finnish, the primary word-level stress is assumed to always fall on the first syllable of each word (Hakulinen et al., 2004; Karlsson, 2013), experimentally investigated by, for example, Suomi, et al. (2003).

In Finnish, the common disyllabic units, feet, consist of a first stressed syllable and a following unstressed syllable. Thus, feet can be described as left-headed. The primary word stress can be realized by segments having longer durations as opposed to secondary stress. In Finnish, the primary stress is also phonetically manifested by different pitch contour patterns, with the pitch peak usually being located in the first (stressed) syllable (Suomi et al., 2003). Generally, these kinds of word stress realization patterns result in a falling pitch contour in a foot sequence, in both Finnish and North Sámi (Nickel & Sammallahti, 2011).

**Figure 2.**
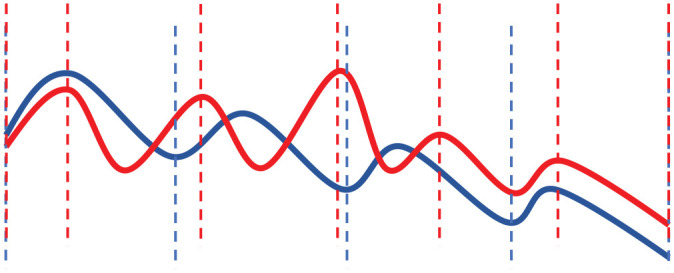
Stylized intonation contours from Norwegian (in red, adapted from Heggtveit and Natvig (2004) and Finnish in blue, adapted from Iivonen (1998); Karlsson (2013)). The vertical lines indicate the word boundaries.See the online article for the color version of this figure. See the online article for the color version of this figure.

Unlike Finnish and North Sámi, Norwegian (a North Germanic Indo-European language) does not have fixed stress on the first syllable, but uses a lexically alternating stress position, often falling on the second syllable (a tendency of right-headedness) (Kristoffersen, 2000). The most prominent accentual phrase in an utterance typically has a higher pitch level at the right edge than the less prominent ones. Importantly, in most Norwegian dialects, there are two lexically distinctive pitch accents, which together with the phrase accent patterns form relatively active intonational contours with smaller declination than in Finnish (Heggtveit & Natvig, 2004; Wetterlin, 2010) (see Figure 2). The northernmost dialects of the Bokmål Norwegian that are in contact with North Sámi are characterized as “high-pitched” or *høytone* (Nickel & Sammallahti, 2011), meaning that the syllable stress is marked by a pitch peak. Consequently, if the word stress of a disyllabic word is on the second syllable, the F0 contour within the word is rising, unlike in Finnish and North Sámi (see examples in Nickel & Sammallahti, 2011). Importantly, Norwegian has a lexical pitch accent (or *lexical tone*), which means that many segmentally homonymous bisyllabic word pairs differ only in their tonal contours: *falling* or *rising-falling* (Wetterlin, 2010), for example, *aksel1* “shoulder”; *aksel2* “axle.”

Compared to Norwegian, the Finnish intonation patterns are relatively stable and more uniform, with gradually falling intonation over the entire utterance, in general. Following Iivonen (1998), a basic declarative and non-affective utterance consisting of several words manifested prosodically as a descending F0 curve, including rising–falling peaks on the stressed syllables, or “. . . a succession of declining rising–falling patterns (on content words), with an end reaching a very low F0 level (eventually containing non-modal phonation)” (Suomi et al., 2008, p. 115). In most cases, a declination of F0 can be observed, as shown in Figure 2. However, word prosody is inevitably influenced by utterance prosody and the different focus conditions. Although the utterance intonation in Finnish is relatively steady, new information can be signaled by a greater pitch excursion (Arnhold et al., 2010; Karlsson, 2013; Suomi et al., 2008). The speech data used in this work consist of declarative read speech (with nearly the same text read aloud by all speakers), and we focus on word level prosodic patterns. In what follows, we describe the materials and methods used in this study.

## 5 Materials and methods

### 5.1 The extended DigiSámi corpus

To analyze the prosodic differences between the North Sámi areal varieties and mapping the majority language influence, we used the DigiSámi read speech corpus (Jokinen & Wilcock, 2014). It was collected from five locations traditionally inhabited by the Sámi: Anár, Avvil, and Ohcejohka in Northern Finland, and Guovdageaidnu and Kárášjohká in Northern Norway (see Figure 1). The DigiSami corpus is available via the CSC website (www.csc.fi) by contacting the author of the original data (Jokinen, 2018).

The task of the speakers was to read aloud Wikipedia articles about the Sámi languages and traditions. The corpus consists of speech data from 25 native (16 females) North Sámi speakers, with ages ranging from 16 to 65. The data were recorded using a 4-channel portable recorder (Roland Edirol R-4 Pro) and a condenser mini-lavalier microphones (AKG C 417L). The speech data also originally contain sentence level annotations—for a more detailed description of the data collection and corpus, see Jokinen, 2014, 2018; Jokinen et al., 2017). According to the questionnaire information described by Jokinen (2018), most speakers used North Sámi as the main language of communication when interacting with other people and there were no remarkable differences in the language skills between the participants.

To extend the original DigiSámi speech corpus, an additional corpus (the Extended DigiSámi corpus) with nearly the same texts read by different speakers was collected by the authors in 2018 in Guovdageaidnu and Oulu, following the ethical guidelines of the University of Helsinki. There were altogether 148 individual sentences in the text material of the Extended DigiSámi corpus. Speech data from seven (four females) more speakers was added to the Extended DigiSámi corpus (age range 22–64). The five speakers recorded in Guovdageaidnu were all from Guovdageaidnu except one, originally from Kárášjohká. The speakers recorded in Oulu were originally from Ohcejohka and Avvil. To make the two corpora as compatible as possible, the recording process followed similar protocol as in the Extended DigiSámi corpus (Jokinen & Wilcock, 2014), with the inclusion of some additional sentences of the same reading topics. The recordings were done in small and quiet office rooms and a Zoom H2n portable recorder with five built-in microphones was used (Mid-Side recording mode). Most of the speakers of the Extended DigiSámi corpus did not consent to have their speech data publicly available (outside the University of Helsinki), and we respect their wish not to publish it.

Altogether, the Extended DigiSámi corpus contains material from 31 native North Sámi speakers and one fluent non-native. It is important to note that all North Sámi speakers are bilingual: the speakers from Guovdageaidnu and Kárášjohka were also native in Norwegian, while those from Ohcejohka, Avvil, and Anár were bilingual in Finnish and North Sámi.

In terms of the dialectal varieties, only the Guovdageaidnu areal variety belong to the Western dialect area according to the traditional analysis of North Sámi (see Sammallahti, 1998). The rest of the varieties belong traditionally to the Eastern dialect group of North Sámi (see Figure 1). In Guovdageaidnu and Kárášjohká, there are exceptionally large and active Sámi communities and North Sámi is spoken by the majority of the inhabitants.

The original Extended DigiSámi data were previously manually annotated and segmented on sentence level. For this paper, we further annotated the entire Extended DigiSámi speech data semi-automatically at the word and segment levels, using the WebMAUS Basic forced alignment tool (Kisler et al., 2017). Each word was also POS tagged. Additionally, the sentences were divided into phrases, following the natural syntactic structures of North Sámi, resulting in approximately three phrases per sentence. Note that this division was done on the textual material and only subsequently applied to the speech signal, therefore these phrases do not necessarily correspond to intonational phrases. Please refer to Appendix B for examples of the sentences and their division. All labels and segmentations were manually checked and corrected after any automatic procedures. At this point, however, we did not label for focus; there is unfortunately no research on North Sámi focus, and establishing a reliable framework for focus labeling would require its own, separate research contribution. The aim of our machine learning technique is to explore the data using as little manual annotation as possible.

Due to varying recording circumstances and certain voice quality characteristics of the speakers, we used a subset of the speech data described above. Unfortunately, one-third of the speakers (10 of 31) and a number of sentences (79) from the remaining speakers had to be excluded because of hoarse/creaking voice, hesitations, mistakes or severe background noise overlapping with normal speech frequencies. A sentence was discarded from the analyzed data if there were more than 10 errors in the F0 analysis (mostly octave jumps).

Two older speakers from Guovdageaidnu, excluded from the analysis, had a hoarse voice and many of hesitations, which made reliable pitch extraction impossible. The same applied for two cases (one middle-aged and one old) from Anár, Finland, where the voice of a speaker was so consistently hoarse or creaky that there were too many (more than 10) errors in the pitch extraction in nearly all sentences from that speaker. In these cases, the data would not have been reliable in terms of the prosodic analysis and a decision was made to exclude the speaker from the data. A number of speakers (six young speakers from Avvil, Finland) were excluded from the data because of a consistent buzzing noise in the critical speech frequencies which made it impossible to filter out the noise without compromising the actual speech signal.

The details (site, dialect, duration and age groups) of the remaining and analyzed data set are described in Table 1. Altogether, eight speakers from Finland and two speakers from Norway were excluded from the analysis. This resulted in an unbalanced amount of speakers from Finland (nine speakers) compared to Norway (12 speakers) but the difference between the amount of speech data in minutes (107 minutes vs. 114 minutes, respectively) was not considered to be remarkably different in a way that it would distort our analysis.

**Table 1.** Description of the read speech data analyzed in this research. The thick line separates the Finnish and Norwegian sites. The total duration for the Finnish sites was 106:59 minutes and for the Norwegian sites 114:49 minutes. Age groups: Y = young (15–25); M = middle (25–50); and O = old (50–70).

Majority **language**	**Site**	**Dialect**	Speakers **(Female)**	Audio duration **(minutes: seconds)**	Age group **distribution**
Finnish	Anár	East	2 (2)	21:10	1Y 1O
Finnish	Avvil	East	1 (1)	11:32	1Y
Finnish	Ohcejohka	East	6 (1)	64:07	1Y 3M 2O
Norwegian	Guovdageaidnu	West	6 (4)	71:24	1Y 3M 2O
Norwegian	Kárášjohká	East	6 (4)	43:25	4Y 1M 2O
Total			21 (12)	221:48	

### 5.2 Network architecture

**Figure 3.**
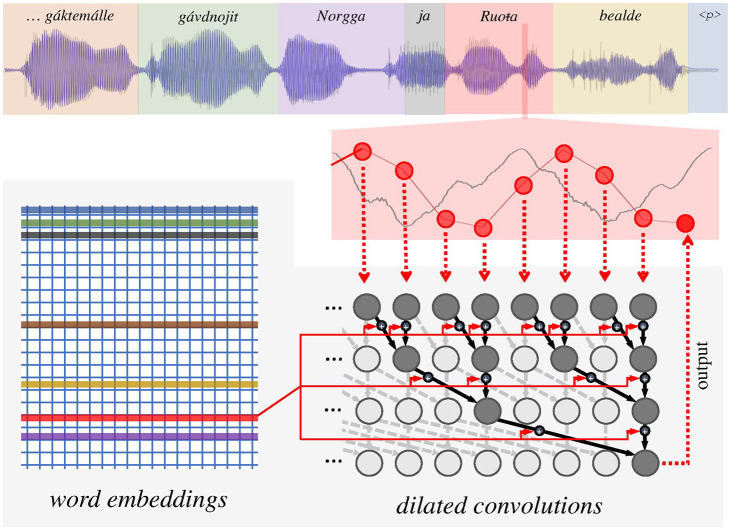
An architecture of the WaveNet synthesis system with the conditioning embeddings using prosodic signal.

The WaveNet (Oord et al., 2016) is a deep artificial neural network speech synthesis architecture that operates directly on the digital (pulse-code modulation) signal. In essence, the network is trained to output an individual signal sample using a preceding portion of the signal samples as input. As illustrated in Figure 3 (bottom right shaded areas) the input samples are processed by several stacked-up dilated convolutional layers. Every convolutional layer contains a number of convolutional filters whose output depends on values of several (two, in the case of this particular architecture) outputs from the previous layer. As shown in Figure 3, at each time-step, every output from the first hidden layer depends on two subsequent input samples. The following hidden layer, however, combines the values from the previous layers that are separated (dilated) by two time-steps, rather than subsequent values; each value thus depends on four input samples. The convolutional filters on the next layer are then dilated by four time-steps, and each output value is a function of eigth input samples.

This architecture facilitates an exponential increase of the length of receptive field (the previous portion of the input signal that determines the value of every given output sample) with every stacked convolutional layer, and provides a sort of parallel hierarchical analysis with more dilated layers capturing progressively longer-term dependencies in the signal.

In the present work we used a TensorFlow (Abadi et al., 2016) implementation of the WaveNet network architecture with two stacked-up sets each containing nine stacked layers with dilations incrementally doubled for each subsequent layer: 1, 2, 4, . . ., 256, 1, 2, 4, . . ., 256 (repeating several identical stacks of appropriately dilated convolutions does not increase the receptive field size, but is a common practice). This leads to the receptive field of length 1024 samples. See Appendix A for a technical description of the network used in this work.

The receptive field size determines the maximal temporal scope of dependency that can be learned by the network. As we are aiming to use our WaveNet implementation for a prosodic analysis, we want to be able to capture dependencies between events separated by at least several hundreds of milliseconds. While the receptive field size is a function of the number of hidden convolutional layers, its temporal scope obviously also depends on the sampling rate of the signal. Therefore, we trained the network on a low sample rate prosodic signal created from the high sample rate original speech signal.

### 5.3 Pre-processing and prosodic signal generation

As we aim at the analysis of the speech material in terms of prosody (rather than, e.g., segmental information), a new *prosodic* signal was generated for each sentence matching the original waveform in the F0 and energy envelope but containing no harmonics of the F0. This prosodic signal, designed to capture only the pitch and intensity of the original speech is sampled at a relatively low sampling rate of 800 Hz; this means that the 1024 samples of the receptive field covers 1.28 seconds of speech.

There are several reasons behind generating a completely new signal rather than using a downsampling filtering-based procedure. One was to avoid inclusion of speaker and site specific interference in the automatically analyzed material, such as background noise, room reverberation, etc. Another motivation is to recast the F0 contours of individual speakers to a common baseline (of 100 Hz median F0 for each speaker) while keeping the relative pitch movement intact (see below).

An alternative option satisfying both these criteria would be to train the network on paired low sampling rate F0 and intensity signals. While this approach is certainly viable and should be investigated in the future, in this work we opted for generating prosodic signals instead for two reasons: (a) the WaveNet network architecture has been primarily used and tested on “speech like” signals (intensity modulated carrier frequency signals); and (b) in our experience the architecture does not work well with two parallel signals.

The prosodic signal was created by the following procedure.

The F0 was extracted using a customized interactive Praat (Boersma & Weenink, 2016) script, allowing for manual checking and correcting octave jumps, and labeling creaky phonation, noise. and other artifacts. Interpolated contours were used for unvoiced intervals as well as for the intervals with identified creaky phonation. The resulting F0 contours were sampled at 800 Hz. Identically sampled (at 800 Hz) energy envelope e was also extracted from the signal by filtering a square of the original waveform signal filtered by a 25 milliseconds Hamming window.

In order to remove the pitch differences among speakers caused by natural physiological variability (leading to, e.g., differences between males and females) the pitch contours were modified so that the median F0 for each speaker equaled 100 Hz. The extracted F0 contours were first recast to semitone scale using the median F0 for each speaker (over the entire material) as a base frequency, and then new F0′ signals in Hz scale were recomputed using the base frequency of 100 Hz.

The envelope-modulated sinusoidal prosodic signal was then generated as

$$s_{n}=e_{n}\sin\;(2\pi sr\sum_{i=1}^{n}\;{F0\prime }_{i}),$$

where $e_{i}$ and $F0_{i}$ denote the i th sample of the envelope and F0′ signals, respectively, and sr=800 is the sampling rate^1^.

**Figure 4.**
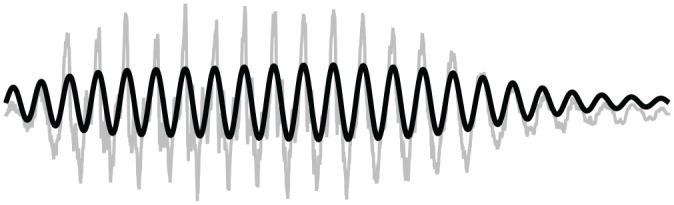
Original audio waveform (in gray) superimposed with a sinusoidal prosodic signal (in black).

As illustrated in Figure 4, without the pitch normalization described above, the signal s has the same F0 and energy envelope as the original, but contains no segmental or spectral information. Due to the pitch normalization, the resulting prosodic signals used here have F0 equal to F0′.

### 5.4 Embeddings

In addition to conditioning by the previous signal, the WaveNet architecture uses (local or global) conditioning to generate a signal with the required characteristics (Oord et al., 2016). The known characteristics of the signal are fed as an additional input to each dilated convolutional layer via embedding layers (see the network connections rendered in red in Figure 3), trained together with the other network components. The embedding layer learns to map a discrete set of control parameters (e.g., a set of individual words in the corpus) to real-number valued vectors that are directly used as an additional input to each convolutional layer (the matrix shown in the left portion of the shaded area in Figure 3). In the case of local conditioning used here, the appropriate embedding vector trained in a particular time is determined by a parallel signal containing the relevant control parameter value corresponding to the trained signal sample. In Figure 3, the different values of this parameter (corresponding to different words in the utterance) are schematically depicted by different coloring of waveform background and the corresponding coloring of the rows of the embedding matrix.

The main aim of the machine learning part of the presented method is to obtain vector representations of individual lexical items as they are produced by, on the one hand, the Sámi speakers from Finland and, on the other hand, by the speakers from Norway. Also, as frequent words might have been produced in multiple sentences and sentence positions, we want to at least to some extent account for possible differences that are attributable to sentence-level prosody.

With this aim, two local conditioning embeddings were implemented in the network architecture and trained in parallel: a *word embedding*; and a *phrase embedding*.

As stated in our methodological hypothesis (MH), the word embeddings are designed to capture the conditioning participating in the generation of the individual lexical items present in a corpus. Using the word-level annotation of the corpus, each sample of the prosodic signal was assigned a label corresponding to the word within which it occurred. For a given word in the corpus, one label was assigned to all renditions of the word by the speakers from Finland, and another label to the renditions by the speakers from Norway. The silent gaps between the words were assigned a separate “placeholder” label (illustrated in light-blue in Figure 3). Only the top 199 most frequent words in the corpus were assigned individual labels; the remaining words were grouped together under two common labels (one for the Sámi speakers from Finland, one for those from Norway). Altogether, 401 ( 1+2×199+2 ) conditioning vectors were trained.

The phrase embedding, referring to a part of an individual sentence in the text corpus, is designed to account for the prosodic variation associated with the position of the given portion of prosodic signal (corresponding to a word) within a particular phrase in a sentence. If the same lexical item occurred in multiple sentences (or parts thereof) its word embedding vector learned the representation of the common aspects of all these realizations, while the phrase embedding vector (different for different contexts) captures the differences associated with the given portion of the sentence.

The labels were assigned using the phrase annotations in the corpus; again separate labels assigned to “Finnish” and “Norwegian” Sámi renditions. The majority of the sentences (altogether 148 individual sentences) in the text materials were divided into approximately three shorter phrases. To follow the natural North Sámi syntactic patterns and to keep the phrase length as comparable as possible, shorter sentences were not divided into phrases at all. Altogether, 284 different phrases were identified in the corpus, resulting in 569 phrase conditioning vectors ( 2×284+1 for the silent gaps between phrases).

Please note that we primarily focus on the word embedding and use the phrase embeddings to automatically account for the occurrences of the given word in different sentences and sentence positions. This decision does not mean that we are not aware of the possibility of prosodic phenomena at phrase-level or beyond. The scope of phenomena that can be investigated using our chosen approach is, however, limited by the length of the receptive field (a bit over one second in the present implementation).

We provide the prosodic signals used in this work, the corresponding word and phrase labeling files as well as the trained embedding as Online Supplementary Material for this article.

To summarize, the task of the network is to learn to predict the next sample of the prosodic signal, given its phrase and word identity, as well as the speaker’s country of origin. In order to generate an appropriate prosodic signal, the fully trained network would receive two parallel sequences of (appropriately aligned) word and phrase labels; as there is no temporal modeling attempted in the present implementation, the word/phrase sequences would need to be of appropriate length. The network would generate a “Norwegian” or “Finnish” signal, depending on the labels (mixing the languages would also be possible by feeding a “Finnish” word label alongside a “Norwegian” phrase label, or vice versa).

Given these input labels (and a default “starting” signal sequence of the receptive field length), the network would generate the signal in an auto-regressive manner, using the previously generated samples as an input.

The WaveNet implementation used in this paper was, however, not designed and tuned to yield a high-quality synthesized prosodic signal. Instead, we focus on the learned word representations derivable from the trained network weights. In particular, we analyze the differences between embeddings for individual words as uttered by the “Finnish” and “Norwegian” Sámi speakers.

### 5.5 Training procedure

All data were used to train the network, as a training set. Obviously, this decision means that the network may overfit the data (fail to generalize to other similar tasks), and that its performance cannot be subsequently tested on a previously unseen test set. This approach would be clearly detrimental if the task was to train a speech synthesizer that can generate signals for previously unseen sequences of words. The aim of this work is, however, to capture the statistical properties of the entire corpus material. Moreover, what is subsequently analyzed is not a generated signal but part of the trained network itself, namely the word embedding layer, that does not change with the input to the network after the training.

A validation set comprised a subset of 20% of the data and was used for stopping the training process: training was stopped after 300 epochs, or earlier if the loss calculated on the validation sub-set did not improve for 25 subsequent epochs.

The training was repeated 10 times, and the word embeddings were extracted after each training run.

## 6 Results

### 6.1 Word embedding distance and comparison of the models

The analysis was limited to the words with at least 17 separate occurrences for each language group in the corpus; 70 words fulfilled this requirement. The number 17 was chosen rather arbitrarily, but it yields a sufficient number of tokens for the analysis and guarantees enough repetitions for each token. Using other cut-off numbers in the vicinity of 17 does not qualitatively influence the subsequent results.

To address our typological hypothesis (TH) and in order to obtain a measure of differences between the prosodic realizations of these words as captured by the trained models, for each word we calculated the distance between the embedding vector for the word for the “Finnish” Sámi speakers from the embedding for the “Norwegian” Sámi speakers. First, each trained embedding matrix was “spherized” by first subtracting the mean from the every embedding vector and then by dividing every column in the resulting matrix by its standard deviation. Second, as it is likely that some of the embedding vector dimensions did not capture any relevant information, we transformed the spherized vectors using a principal component analysis and used only the first 32 dimensions (explaining about 75% of variance) in subsequent analysis. Spherization and principal component analysis were performed using MATLAB.

**Figure 5.**
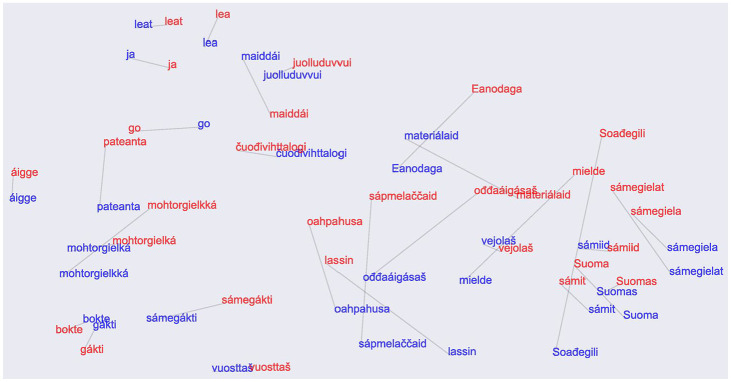
Uniform Manifold Approximation and Projection of embeddings of 30 closest word pairs: red, Norwegian Sámi; and blue, Finnish Sámi.See the online article for the color version of this figure. See the online article for the color version of this figure.

Figure 5 shows the resulting 32-dimensional vectors of one model, recast to a two-dimensional space using the Uniform Manifold Approximation and Projection (UMAP) (McInnes et al., 2020) method. We observe that despite the lack of spectral information in the signal, the phonotactically similar words are generally clustered together in the embedding space. In addition, the Norwegian and Finnish variants of the words are close to each other, but with varying distance. Direction between variants does show some pattern, but there is no general trend between language variants, suggesting that our signal processing procedure successfully mitigated the possible group differences between “Norwegian” and “Finnish” speakers, unrelated to our research question, such as, for example, difference gender/age distributions, potential differences in recording conditions, etc. Note that the distances in this two-dimensional projection do not directly correspond to the actual embedding distances discussed below.

For each word, a Euclidean distance between the pair of 32-dimensional embedding vectors—one for each majority language group—was calculated. In order to verify that the trained models yielded reasonably similar word distance measures, we calculated pairwise Pearson correlation coefficients comparing the word distance vectors for different models. The correlations ranged between 0.80 and 0.90, with a mean of 0.85. Consequently, we averaged the word distances for each word across all 10 trained models; these mean distances will be used in the analysis below.

**Figure 6.**
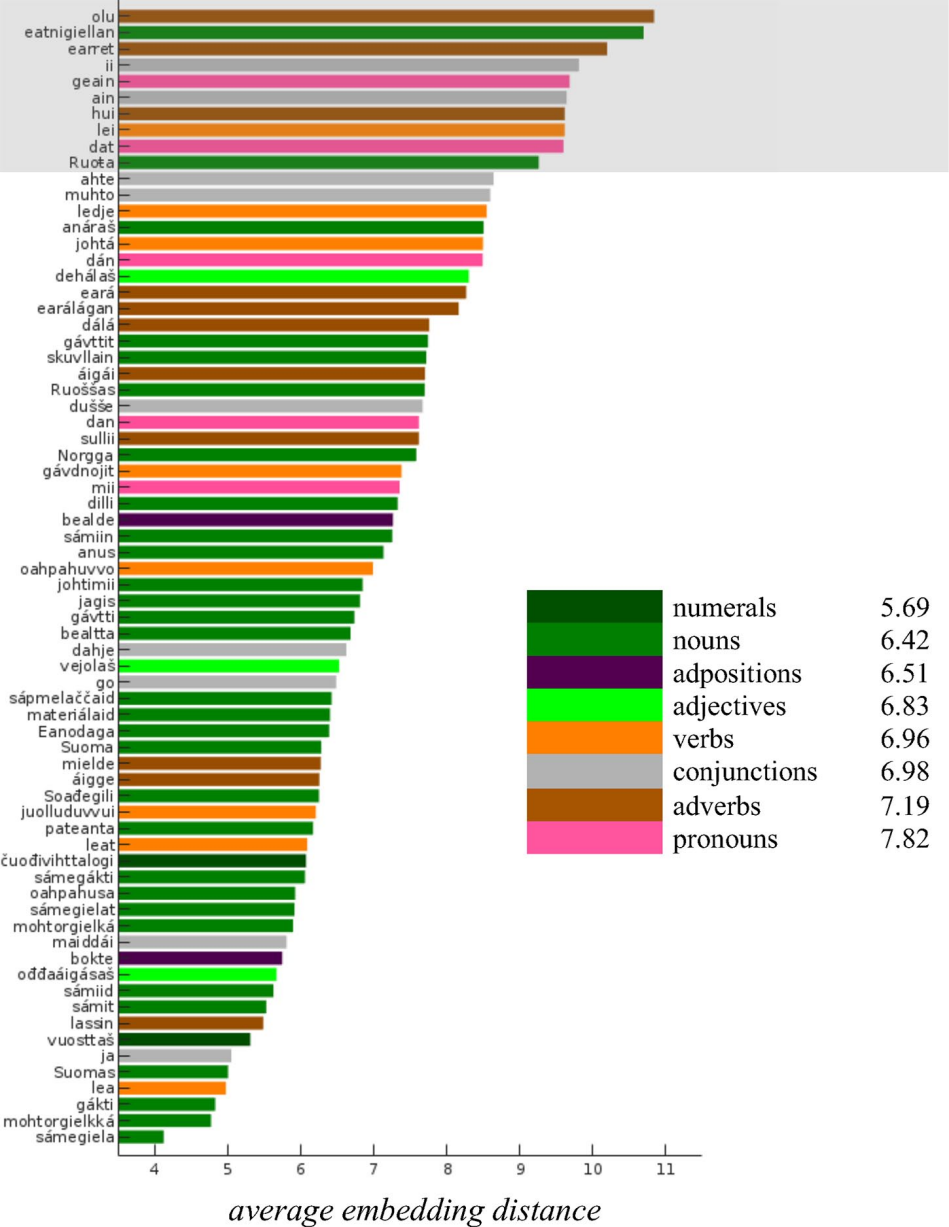
Average embedding distances for the most frequent words in the corpus. Coloring reflects the part-of-speech category of each word. Note that the inflections of the copula (*lea, leat, ledje*) are altogether more frequent in the text than the other verbs which might also reflect in the prosodic renditions of these words.

The barplot in Figure 6 shows the embedding distances between the Finnish and Norwegian realizations for the 70 most frequent words in the corpus. Note that for the top 10 words (the portion with the gray background from “*olu*” to “*Ruota*”), the distances are somewhat greater than for the remaining words (see the “offset” between “*Ruota*” and “*ledje*”). An inspection of the speech material revealed that this is probably caused by frequent hesitations and corrections within these words in the corpus; therefore, these words were excluded from the subsequent analysis.

The bar colors in Figure 6 indicate the POS categories of individual words. Note that the “green” colored words (nouns, adjectives, and numerals) primarily occupy the lower part of Figure 6, while verbs, adverbs, adpositions, and pronouns tend to occur in the upper portion. This observation is supported by the mean embedding distances for different POS categories listed as part of the legend in Figure 6.

### 6.2 Comparing word distance with F0 characteristics

Can the differences between the Finnish and Norwegian Sámi renditions captured by the embedding distances be associated with some particular characteristics of the prosodic signals (as also formulated in our methodological hypothesis (MH))? In order to address this question and the MH we extracted two F0-related measures from the original corpus, namely the mean F0 range and mean F0 standard deviation (*SD*) over the Finnish and Norwegian renditions of each analyzed word.

More precisely, given a word, corresponding F0′ (the median-normalized pitch) values were extracted for every occurrence. Then, standard deviation of these values was calculated, and these standard deviations were averaged across all occurrences for Finnish speakers and all occurrences for Norwegians, yielding the group specific *mean* F0 *SD* for the word. Similarly, the differences between 90th and 10th percentiles of the F0′ values were used to calculate the group-specific *mean* F0 *ranges* for each word.

Finally, the difference of these two measures between Finnish and Norwegian speakers were calculated. F0 *SD distance* for a word is the Finnish mean F0 *SD* minus Norwegian mean F0 *SD* for the word. Similarly, F0 *range distance* is the Finnish mean F0 range minus Norwegian mean F0 range for the same word.

When all 60 words are taken together, neither of these distance measures significantly correlates with the embedding distance: for the *SD* distance, the Pearson correlation is 0.16 ( p=0.21 ), and for the range distance the correlation is 0.05 ( p=0.72 ).

The situation is somewhat different when the words are grouped by their POS categories. Table 2 shows correlations between the *SD* distance and range distance, respectively, and the embedding distance for different types of words. While in most cases the correlation coefficients failed to reach significance (presumably due to relatively low counts; column *n* in Table 2), the correlations reveal an interesting pattern.

**Table 2.** Correlations between the embedding distances and the acoustic distance measures, for different part-of-speech (POS) categories.

POS	*n*	Mean fundamental frequency (F0) standard deviation		Mean F0 range	
		Coefficient	*p* value	Coefficient	*p* value
Nouns	28	**0.38**	**0.045**	0.25	0.209
Adjectives	4	0.28	0.727	−0.07	0.932
Conjunctions	7	0.46	0.299	0.48	0.275
Verbs	7	−0.53	0.220	−0.57	0.186
Adverbs	7	−0.68	0.095	−0.64	0.119
Pronouns	3	−0.63	0.564	−0.70	0.505
“Noun” group	34	**0.43**	**0.012**	0.23	0.196
“Verb” group	19	−**0.57**	**0.012**	−**0.57**	**0.010**

The coefficients and the respective *p* values marked in bold are considered as statistically significant (*p* < 0.05).

The correlations are positive for nouns, adjectives, and conjunctions, indicating that the greater embedding distance is associated with greater F0 movement (in terms of *SD* and range) for Finnish renditions than for Norwegian ones for these categories. For the remaining POS types, the correlations are negative, meaning that for the most distant words (in terms of embeddings) the pitch excursions are larger in the words uttered by the Sámi speakers from Norway compared to the ones from Finland.

The bottom two rows in Table 2 list the correlations for two groups of words: the first, “noun” group containing nouns, adjectives, and numerals; and the second, “verb” group containing verbs, adverbs, adpositions, and pronouns. With the exception of the correlation between range and embedding distances for the “noun” group, the correlation coefficients are significant for the grouped word categories. Note that we use the group names “*noun group*” and “*verb group*” as shorthand names for the groups since they mostly contain nouns or verbs and words that have similar syntactical roles in North Sámi, respectively.

**Figure 7.**
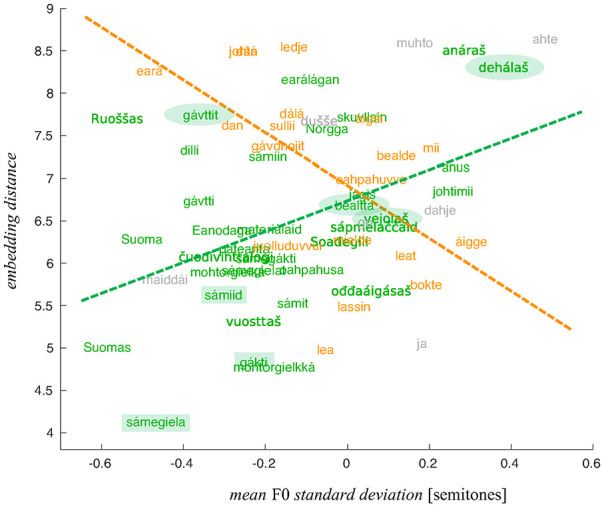
Embedding distance of words as a function of the mean fundamental frequency standard deviation distance. The “noun” group in green, the “verb” group in orange, the conjunctions in gray.

**Figure 8.**
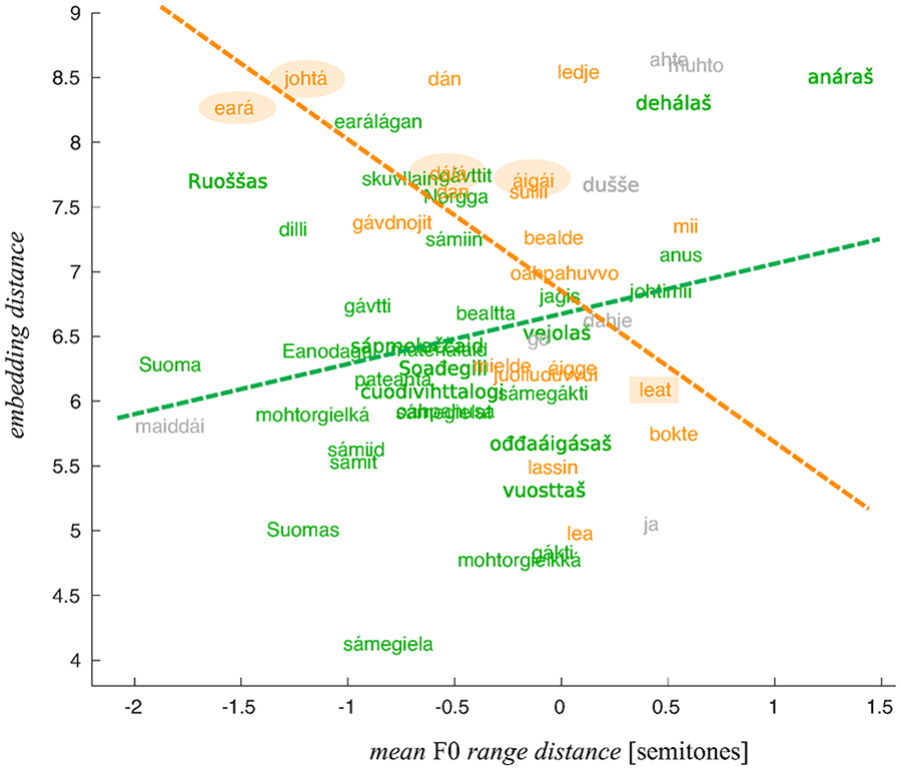
Embedding distance of words as a function of the mean F0 range distance. The “noun” group in green, the “verb” group in orange, and the conjunctions in gray.

This observation is illustrated in Figures 7 and 8 showing the relationships between the *SD* distance and range distance, respectively, and the embedding distance. The coloring of the words indicates their belonging to the two POS groups and the linear fits show the nature of the relationships between the distance measures obtained using the WaveNet embeddings and more traditional acoustic measures.

### 6.3 Comparing F0 contours for individual words

Figures 5–8 can be used to inform us about which individual words might show the clearest differences between the two majority language groups. Of course, the greatest embedding distance does not automatically mean that the given word provides the best example of prosodic phenomena of interest. The link between the quantified intonation phenomena and embeddings is indirect: the differences between the “Finnish” and “Norwegian” renderings are, presumably, signaled by relatively great distances between the respective embeddings, and these, in turn, correlate with *SD* distance and range distance. The fact that these measures correlate does not necessarily mean that *every* word yielding a great embedding distance will also manifest the relevant phenomena in an interpretable way.

In what follows, we present a more detailed comparative analysis of several words (from the two POS category groups) with a considerable embedding distance (circled in Figures 7 and 8) and compare them with words that yielded the smaller embedding distances (rectangles in Figures 7 and 8). The words that were selected illustrate the link between greater F0 range/*SD* distances and the embedding distance in the most straightforwardly interpretable way.

**Figure 9.**
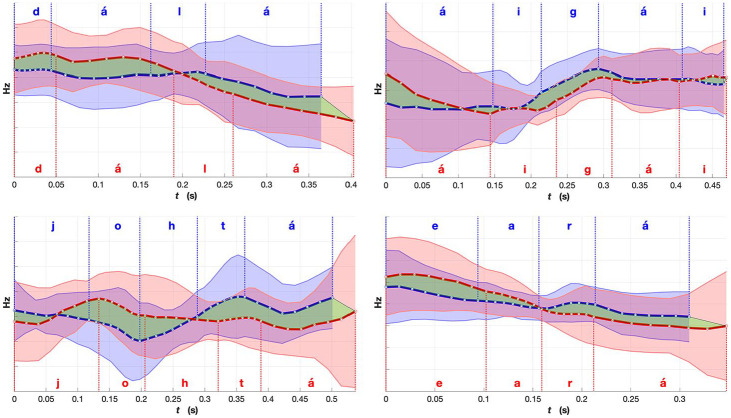
Comparison of mean fundamental frequency (F0) for words from the “verb” group (verbs, adverbs and adpositions): red, Norwegian Sámi; and blue, Finnish Sámi. Shaded region indicates standard deviation. The green region emphasizes the differences in the mean F0 between the two averaged renditions of the words. The word meanings, part-of-speech labels and expected placement of lexical stress (in bold): *d* ***á****lá* ***ái****gái*—“until nowadays” (adverb); *j* ***o****htá*—“to pass/carry” (verb); and ***ea****rá*—“other” (adverb). The F0 values are plotted in Hz scale and created using the range and time normalized F0 contours.See the online article for the color version of this figure. See the online article for the color version of this figure.

**Figure 10.**
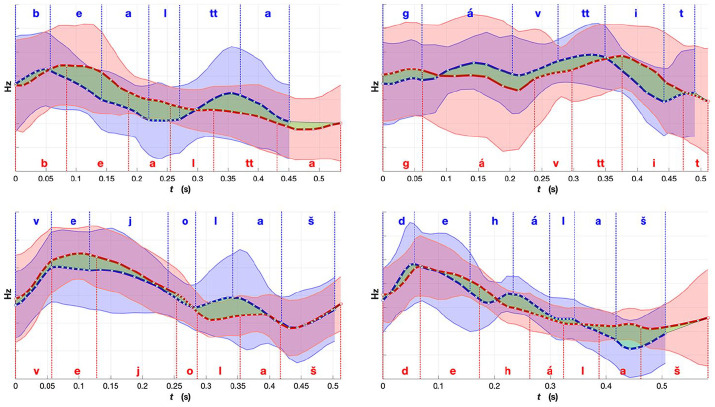
Comparison of mean fundamental frequency (F0) for words from the “noun” group (nouns and adjectives): red, Norwegian Sámi; and blue, Finnish Sámi. Shaded region indicates standard deviation. The green region emphasizes the differences in the mean F0 between the two averaged renditions of the words. The word meanings, part-of-speech labels and expected placement of lexical stress (in bold): *b* ***ea****ltta*—“a belt” (noun); *g* ***á****vttit*–“the Sámi costumes” (plural, noun); *v* ***e****jolaš*—“possible” (adjective); and *d* ***e****hálaš*—“important” (adjective). The F0 values are plotted in Hz scale and created using the range and time normalized F0 contours.See the online article for the color version of this figure. See the online article for the color version of this figure.

**Figure 11.**
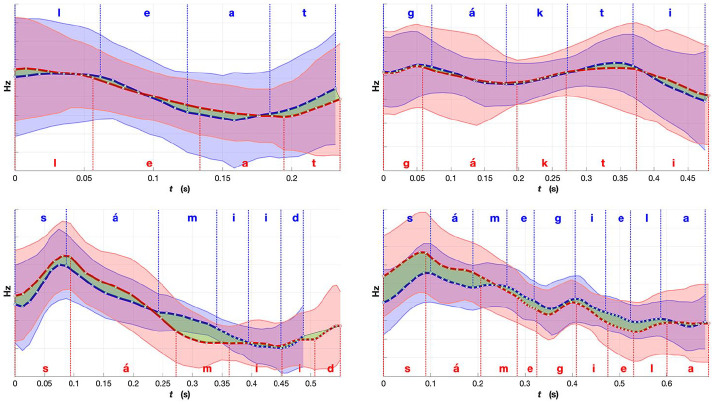
Comparison of mean fundamental frequency (F0) for words with similar prosodic realizations: red, Norwegian Sámi; and blue, Finnish Sámi. Shaded region indicates standard deviation. The green region emphasizes the differences in the mean F0 between the two averaged renditions of the words. The word meanings, part-of-speech labels and expected placement of lexical stress (in bold): *l* ***ea****t*—“to be” (copula, verb); *g* ***á****kti*—“a Sámi costume” (noun); *s* ***á****miid*—“the Sámi people” (plural genitive, noun); and *s* ***á****megiela* —“the Sámi language” (singular genitive, noun). The F0 values are plotted in Hz scale and created using the range and time normalized F0 contours.See the online article for the color version of this figure. See the online article for the color version of this figure.

For selected words we calculated the mean F0 contours and standard deviation bands, separately for the ”Norwegian” and ”Finnish” North Sámi speakers. Using the segment-level annotations, mean segment durations were calculated for a given word’s renditions by Sámi speakers from Norway and those from Finland. These average segment durations were used for time-normalization, with every segment divided into seven equally spaced intervals. Mean F0 values and standard deviations at the corresponding interval boundaries were calculated using the speaker-normalized F0′ contours, and time-warped to the corresponding points in the average segment intervals. The resulting time-normalized and speaker-normalized pitch contours and standard deviation bands are plotted in Figures 9–11; the blue and red corresponding to ”Norwegian” and ”Finnish” Sámi participants, respectively; the vertical lines mark (average) segment boundaries; and the white circles mark the time-normalization points. For all of the selected example words in Figures 9–11, *the y-axis shows the normalized mean F0 in Hz, and the x-axis indicates the normalized time in seconds*. The sentence contexts of the example words are shown in Appendix B. Senrence contexts are also shown in Appendix B.

Figure 9 shows examples of mean F0 contours (time aligned and speaker-normalized) from the “verb” group including verbs, adverbs, and adpositions. In this category, there were certain different patterns in the Norwegian and Finnish Sámi areal varieties. All of the four example words are disyllabic, and in the Norwegian variety, the highest F0 peaks of the word tend to occur within the first syllable. Contrastively, in the Finnish variety, there is a valley in the first syllable, and the F0 rises towards the second syllable and towards the beginning of the next word, likely a noun. Generally, it can be noted that for the Norwegian variety, there is more movement in F0 compared to the Finnish one. The two adverbs plotted in the top panels of Figure 9, *dálá* and *áigái*, forming a phrase *dálá áigái* (meaning “until nowadays”); note the U-shaped overall F0 contour for the Finnish rendition and the more complex Norwegian pattern with a potential peak in the first syllable of the second word.

In the contours in Figure 10, including nouns and adjectives, the F0 of the example words have more movement (or greater range) for the Finnish variety than the Norwegian variety, particularly in the latter part of the words. In Finnish, the first syllable has always the primary stress while the second or third syllable bears a secondary stress (Suomi & Ylitalo, 2004; Suomi et al., 2003, 2008, pp. 75–78). If nouns are more prominent in terms of F0 range or movement (two peaks), the latter F0 peak could potentially correspond to the secondary stress in Finnish North Sámi renditions. In these examples, the timing of the peaks are different in the varieties, especially in the disyllabic words (*bealtta* and *gávttit*). Interestingly, both varieties have the highest peak in the beginning of almost all of these examples, differing from the examples in Figure 9. As a word of caution, it has to be noted that two F0 peaks in the average contours do not necessarily indicate two peaks in individual renditions; they may also result from averaging over two distinct patterns (one with an early and one with a late peak) for the particular example.

The differences between the example words in Figures 9 and 10 might look relatively small, but when comparing them with the similar ones in Figure 11, certain patterns can be observed. As the language is the same, with speakers from different areas, radical differences are not expected. What we are looking for are small prosodic differences that could be traced back to the prosodic characteristics of the majority languages. The translations of the sample words in Figures 9-11 as well as their sentence contexts are also shown in Appendix B. In Figure 11, there are examples of some of the most similar words between the two varieties. The F0 contours follow mostly the same directions in not only the disyllabic but also in the 4-syllabic example word. The unifying factor for three of these words is that they are connected to the Sámi culture and are presumably used frequently by the speakers, regardless of the areal variety. One of the examples, *leat*, is a copula, which means it is a very frequent word and it often gets considerably segmentally reduced (e.g., from /leaht/ to /la/) and thus its prosodic rendition might assimilate more to the prosodic patterns of the surrounding words. When considering the duration of all example words (the x-axis), it seems that the Norwegian variety is generally slightly longer in all of the example words.

## 7 Discussion

We presented an example of typological research that uses machine learning for exploratory comparative analysis of prosodic features in terms, in this case, of potential influence of majority language on North Sámi varieties.

The work has been guided by two hypotheses.

The *methodological hypothesis* postulated that the complex statistical models obtained through machine learning capture some *measurable* differences between word-level prosody of the two majority language groups. We have shown that the embedding distance correlates with two phonetic measures of F0 (*SD* and range) but only in a “piecemeal” fashion: the way the greater embedding distance captures differences in word-level intonational patterns depends on the POS category of the words.

The POS categories fall to two groups. The words in the “noun” group (nouns, adjectives, and numerals) are content words that generally tend to have focused acoustic realization, in particular in non-narrow focused read speech. The “verb” group words (verbs, adpositions, pronouns, but also adverbs) tend to be generally more predictable from the surrounding context, and might thus not have as prominent acoustic realization, that is, with less energy, and also less F0 movement (cf. Arnhold et al., 2010). In other words, it seems that in an encyclopedia-like declarative text (delivering new information to the reader/listener), nouns would be discourse-new and therefore prosodically more prominent (e.g., getting pitch accent). These are, however, generalizations about the POS categories in this particular North Sámi read speech corpus, based on the prosodic properties of the most frequent words in our material.

While we did not take the verb type into account in the analyses of the present paper, the presence of accent on verbs has been discussed in the literature as depending on the kind of verb, for example, transitive versus intransitive (transitive verbs in Arnhold et al., 2010, for example). In the most frequent words of our read speech material (see Figure 6), there are, for example, many different inflectional forms of the copula *leat* (*ledje, lei*, and *lea*), but also transitive verbs such as *juolludit* “to grant something,” *gávdnot* “to find something” or *oahpahit* “to teach something” (in this case the Sámi language in schools). There was also at least one verb that had both transitive and intransitive uses: *johtá*, “to pass/carry something” or “to go/travel.” However, the number of tokens per verb (type) would have been presumably too small to be able to conduct any statistically meaningful comparison between the types.

Our results indicate that at least for the words realized most differently between the Finnish and Norwegian bilinguals, the Sámi speakers from Finland produce the “noun” group words with relatively greater, and the “verb” group words with relatively smaller F0 excursions compared to their Norwegian counterparts. The Finnish–North Sámi bilingual speakers thus, presumably, realize the prominence differences between the two word categories to a greater extent, and more systematically than Sámi speakers from Norway.

As we are unable to compare our results with any previous studies on North Sámi prosody, we cannot say that the POS coefficient groupings (the “noun” vs. “verb” groups) would be purely an accident of properties in the corpus, but at least for this particular kind of read speech material, the groupings might show some more general although hypothetical guides on the prosodic characteristics of North Sámi spoken in Finland vs. Norway, potentially a result of the long and intensive contacts between the majority languages and North Sámi.

**Figure 12.**
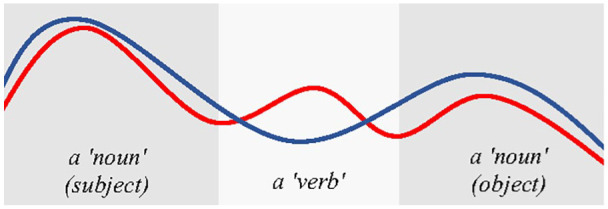
A schematic depiction of possible phrase intonation patterns by Finnish (blue) and Norwegian (red) Sámi speakers for a simple subject–verb–object sequence compatible with our findings.See the online article for the color version of this figure.

This interpretation can be illustrated in a schematic way by the two F0 contours drawn in Figure 12, capturing a realization of a simple phrase (e.g., subject–verb–object). While the Norwegian readers mark each word intonationally, the Finnish Sámi speakers might, hypothetically, reduce the intonation in the “verb” portion and thus have a different peak alignment in a phrase compared with the Norwegian Sámi renditions. This is consistent with the findings of less F0 variance in verbs compared to content words in the Finnish language (Arnhold et al., 2010).

This does not imply that the intonation patterns produced by the Finnish participants are overall more pronounced than those by the Norwegian ones (c.f. Figure 12). As shown in Figures 7 and 8, most of the word-based average differences between F0 ranges and *SD*s are in fact negative. Given the way this measure was defined (Finnish–Norwegian) this indicates a tendency of greater F0 movement for the Norwegian group of speakers than for the Finns for most words, not only “verbs.” This is consistent with the perceived “singing quality” of Norwegian language (Heggtveit & Natvig, 2004; Wetterlin, 2010) compared to generally more “subdued” intonation and “slower” movement in F0 in Finnish, as shown also in Figure 2.

This interpretation of the findings lends some support to our second, *typological* hypothesis postulating that the greatest differences between “Finnish” and “Norwegian” renderings (as identified by the embedding distances) are attributable to the majority language influence and show some similarity with the respective majority language prosody. We, of course, do not claim that the hypothesis is thus confirmed. Rather, we present this interpretation as a new hypothesis that emerged from our exploratory analysis, which needs to be tested in the future using targeted speech material and, potentially, a newly developed phonological description of North Sámi intonation. Importantly, the development of this platform can be, at least partially, informed by our findings.

The targeted speech material should be designed to magnify the majority language effects in terms of differences between “Finnish” and “Norwegian” North Sámi speakers. As can be seen in Figures 9 and 10, the differences between the average renderings by these speaker groups are rather small, and might fail to reach significance if evaluated directly by more standard statistical methods. This, in our opinion, highlights the benefits of using powerful deep learning techniques to generate statistical models (using distributed rather than direct representations of investigated phenomena) that are capable of bringing even this type of subtle influence to our attention for future evaluation.

Regarding the speech material used in the present work, we must consider certain factors about read speech that do not depend on the majority languages or their prosody, and it is important to mention that the hypothesized prominence patterns might also result from other sources. Namely, as the modern North Sámi written standard has been in use only since 1979 (Aikio et al., 2015), there is a possibility that not all readers of all ages are used to reading North Sámi texts, especially if they have not received education in this language. For example, it is possible that the speakers from Finland are—at least on average—more fluent readers than the participants from Norway, possibly because Norwegian and North Sámi are structurally more different languages than Finnish and North Sámi. This would mean that there are more hesitations in the Norwegian material and that these hesitations resulted in more frequent chunking of speech to intonational phrases. Additionally, there is no “spoken standard” for North Sámi, instead, every speaker needs to adapt the reading of written language to her or his own spoken dialect and variety. This is also a potential source of hesitations and repetitions in the spoken material that influences the reading prosody.

In fact, the analyzed corpus contains many examples of these types of sources of variance. While some of this variance could be addressed by the present method (e.g., the uneven distribution of participants’ sex, creaky phonation, variable recording quality, influence of word position within a phrase, etc.), other influences are reflected in generally small sizes of effects as presented here.

The typological interpretations and hypotheses presented in this work are somewhat exploratory and preliminary, and will need further investigation. We have mentioned a new speech material designed to directly address our new hypotheses. Additionally, a perception experiment could be designed and conducted with native speakers of North Sámi, to reveal more details on if and how the characteristics of the different spoken language varieties are being recognized and discriminated.

The work nevertheless illustrates that our methodology can be used for typological analysis, and can generate hypotheses not straightforwardly accessible by a more standard phonetic analysis. As we show, it can be used to analyze speech material not necessarily very suitable for the given comparative analysis. Moreover, the methodology is designed to minimize the need for manual processing of the speech material; for example, it does not require costly ToBI annotation or any other labeling (e.g., focus marking) that cannot be relatively easily automatized. This makes it eminently suitable for exploratory analysis of potentially even “found” speech material from under-resourced languages.

North Sámi is a minority language with its speakers widely dispersed and bilingual in Sámi and one of the majority languages. Our results suggest that the features of majority languages, not present in the North Sámi orthography, are to some extent reflected in the prosodic features, reflecting the multitude of the long and strong language contacts between Norwegian, Sámi, and Finnish.

## 8 Conclusions

The embedding distance measure, capturing the prosodic differences in North Sámi word-level prosody between North Sámi–Finnish and North Sámi–Norwegian bilinguals, can be associated with differences in intonation patterns quantifiable using relatively simple phonetic measures on average F0 trajectories. The sign of correlation between the embedding-based and phonetic-based measures, however, depends on the POS category of the words.

The differences between F0 contours over the words identified as realized most differently by the two speaker groups are consistent with the possibility of prosodic transfer from the majority languages to the studied variants.

Overall, the work shows that the proposed machine-learning-based methodology presents a suitable exploratory tool for prosodic typological analysis, in particular for less well resourced and minority languages.

## Supplemental Material

sj-tgz-1-las-10.1177_0023830920983591 – Supplemental material for Comparative Analysis of Majority Language Influence on North Sámi Prosody Using WaveNet-Based modeling Click here for additional data file. Supplemental material, sj-tgz-1-las-10.1177_0023830920983591 for Comparative Analysis of Majority Language Influence on North Sámi Prosody Using WaveNet-Based modeling by Katri Hiovain, Antti Suni, Sofoklis Kakouros and Juraj Šimko in Language and Speech

## Appendix

### A. WaveNet implementation

The implementation of the WaveNet synthesis used in the present work has followed the original description of the architecture as presented by Oord et al. (2016). Note that the network was trained on the prosodic signal sampled at 800 Hz and described in the *Pre-processing and prosodic signal generation* section. A µ-law companding transformation was applied to the prosodic signal to reduce the dynamic range. The network was trained to generate the processed signal quantized to 64 possible values.

Table A1 summarizes the hyperparameters used in our model.

**Table A1.** Summary of hyper-parameters.

*Signal*	
Sampling rate	800 Hz
Quantization steps	64
*Model size*	
Skip channel dim	128
Residual channel dim	32
Word embedding dim	64
Phrase embedding dim	64
Receptive field	1024
*Learning*	
Optimizer	ADAM (a method for stochastic optimization, see Kingma & Ba, 2014)
Initial learning rate	0.001
Schedule	Exponential decay
Dropout probability	0.05

No exhaustive hyperparameter search for the model was performed due to computational concerns and thus the chosen values are somewhat arbitrary. In general, the number of convolutional filters (skip and residual channels) are greatly reduced from typical WaveNet synthesis implementations. This reflects the relative simplicity of the prosodic signal compared to a full band speech signal, and helps combat over-fitting due to a comparatively small amount of training data. Also, as mentioned, our WaveNet model is trained to predict the next sample at each time-step, given the previous 1024 samples of the prosodic signal and the two conditioning signals. The previous samples provide a strong prior, and thus the model has a tendency to disregard the conditioning input and rely only on the previous samples. Reducing the number of model parameters while keeping the embedding dimensions comparatively high counters this tendency.

The discrete conditioning inputs (phrase identity, word identity + majority language) are projected to continuous high dimensional vectors via embedding layers. The two embedding layers are then connected to each dilated layer via a gating mechanism (as per Equation 5 in Oord et al., 2016), helping the model to make use of the conditioning on appropriate time scales. Dropout was applied on each dilated layer before the embedding connections.

### B. Examples from the read speech text corpus

This section and Table A2 contain examples of phrases/utterances with their English translations, used for training the phrase embeddings. Some of the example utterances are a part of a longer sentence (these utterances are marked with “. . .”). Shorter sentences were not divided into smaller partitions, as they were as long as the utterances divided from the longer sentences. The example Table A2 also shows the phrasal contexts of the example words shown in Figures 9–11. The original texts are sourced from the North Sámi Wikipedia (see Jokinen and Wilcock, 2014).

**Table A2.** Examples of phrases/utterances with their English translations, used for training the phrase embeddings.

Sample word	Sentence context
*dálá áigái* “until nowadays”	*. . .mii lea seilon sámiid geavahusas botkekeahttá* ***dálá áigái***
	“which the Sámi people have used continuously until nowadays”
*johtá* “to pass, to carry”	*Gávtti bokte* ***johtá*** *olu kultuvrralaš ja servodatlaš diehtu.*
	“The Sámi costume carries a lot of cultural and communal information.”
	*Mohtorgielká* ***johtá*** *ovddos- dahje maŋosguvlui bealtta veagas.*
	“A snowmobile moves forwards or backwards using a belt.”
*eará* “other”	*. . .mii ii leat seamma áitatvuloš go* ***eará*** *sámegielat*
	“. . .which is not as endangered as the other Sámi languages”
	*. . .dego silkki, láđđi, silbba ja* ***eará*** *metállaid*
	“. . .like silk, woven fabric, silver and other metals.”
*bealtta*	*. . .mat johte* ***bealtta*** *veagas bensenmohtora fámuin.*
“a belt”	“. . .that move on a belt powered by a petrol engine.”
	*Mohtorgielká johtá ovddos- dahje maŋosguvlui* ***bealtta*** *veagas*
	“A snowmobile moves forwards or backwards using a belt.”
*gávttit*	*Dievddu ja nissona* ***gávttit*** *leat iešguđetláganat.*
“the Sámi costumes”	“The Sámi costumes of a man and a woman are different.”
	***Gávttit*** *hábmejit iežaset gáktemállejoavkkuid.*
	“The Sámi costumes form different groups of designs.”
	*Vuosttas* ***gávttit*** *ledje gorrojuvvon náhkis.*
	“The first Sámi costumes were made of leather.”
*vejolaš*	*Sámegiela lea* ***vejolaš*** *čállit studeantadutkosis eatnigiellan.*
“possible”	“It is possible to take a matriculation test on the Sámi language
	as a mother tongue.”
	*Oalgeávnnasin sámegiela lea* ***vejolaš*** *studeret Helssega universitehtas*
	*ja Lappi universitehtas.*
	“It is possible to study the Sámi language as a minor subject in
	the Universities of Helsinki and Lapland.”
*dehálaš*	*. . .ja danin* ***dehálaš*** *oassi sámiid kultuvra.*
“important”	“. . .and, therefore, an important part of the Sámi culture.”
	*Dán áigge gákti leage coggojuvvon dábálepmosit ávvudemiide*
	*ja* ***dehálaš*** *dilálašvuođaide.*
	“Nowadays the Sámi costume is usually worn as a celebration costume
	and to important occasions.”
*leat*	*. . .mii ii* ***leat*** *seamma áitatvuloš go eará sámegielat.*
“to be (plural copula)	“. . .which is not as endangered as the other Sámi languages.”
	*Sámit* ***leat*** *ain juo guovttegiellagat.*
	“The Sámi people are at least bilingual.”
	*Dievddu ja nissona gávttit* ***leat*** *iešguđetláganat.*
	“The Sámi costumes of a man and a woman are different.”
*gákti*	*Ná* ***gákti*** *bázii vehážiid ávvudanbottuid čiŋađanbivttasin.*
“the Sámi costume”	“So, the Sámi costume remained as a celebration costume
	to infrequent festive moments.”
	***Gákti*** *(suomagillii: lapintakki; dárogillii: kofte) lea sápmelač aid čeardabivttas.*
	“The Sámi costume (. . .) is the traditional clothing of the Sámi people.”
	***Gákti*** *sáhttá muitalit geavaheaddji soga, gili*
	*dahje guovllu, gos son lea eret.*
	“The Sámi costume can indicate its wearer’s family, village,
	or the area where he/she is from.”
*sámiid*	*Suomas* ***sámiid*** *ruoktoguovllus ássi sámiin*
“the Sámi people”	*lea leamaš riekti geavahit sámegiela virgeolbmuiguin.*
(plural genitive)	“In Finland, the Sámi people living in the traditional Sámi areas
	have had a right to use Sámi with the officials.”
	***Sámiid*** *gárvvut leat máinnašuvvon vuosttas geardde*
	*Tacitusa čállán Germania-girjjis.*
	“The clothing of the Sámi people has been mentioned for the first time
	in the Germania book by Tacitus.”
*sámegiela*	***Sámegiela*** *dilli Norgga, Ruota ja Suoma*
“the Sámi language”	*ja Ruošša bealde lea earálágan.*
(singular genitive)	“The situation of the Sámi language in Norway, Sweden, Finland
	and Russia is different.”
	***Sámegiela*** *lea vejolaš čállit studeantadutkosis*
	*eatnigiellan.*
	“It is possible to take a matriculation exam on the Sámi language
	as a mother tongue.”
	*Suomas golbma universitehta fállet* ***sámegiela*** *oahpahusa.*
	“In Finland, three universities are offering courses on the Sámi language.”

## Notes

1. The formula used is a discrete version of $s(t)=e(t)\sin\;(2\pi \int_{0}^{t}F0(\tau )d\tau )$ that produces signal with instantaneous frequency at time t equal to F0(t) and envelope equal to e(t) .
